# Mapping the Most Common Founder Variant in *RSPH9* That Causes Primary Ciliary Dyskinesia in Multiple Consanguineous Families of Bedouin Arabs

**DOI:** 10.3390/jcm12206505

**Published:** 2023-10-13

**Authors:** Dalal A. Al-Mutairi, Basel H. Alsabah, Petra Pennekamp, Heymut Omran

**Affiliations:** 1 Department of Pathology, Faculty of Medicine, Kuwait University, Kuwait City 13110, Kuwait; 2 Zain Hospital for Ear, Nose and Throat, Airport Road, Shuwaikh, Kuwait City 70030, Kuwait; 3 Department of Pediatrics, University Hospital Muenster, 48149 Muenster, Germany

**Keywords:** primary ciliary dyskinesia, genetics of ciliopathy, *RSPH9*, consanguinity, pulmonary diseases

## Abstract

Introduction: Primary ciliary dyskinesia (PCD) is a congenital thoracic disorder caused by dysfunction of motile cilia, resulting in insufficient mucociliary clearance of the lungs. The overall aim of this study is to identify causative defective genes in PCD-affected individuals in the Kuwaiti population. Methods: A cohort of multiple consanguineous PCD families was identified from Kuwaiti patients and genomic DNA from the family members was isolated using standard procedures. The DNA samples from all affected individuals were analyzed by whole exome sequencing (WES). Transmission electron microscopy (TEM) and immunofluorescent analysis (IF) were performed on samples obtained by nasal brushings to identify specific structural abnormalities within ciliated cells. Results: Here, we present six multiplex families with 11 patients who all presented with typical PCD symptoms. Ten out of eleven patients inherited a 3 bp homozygous deletion of GAA in *RSPH9*, whereas the eleventh patients inherited this variant in trans with a frameshift deletion in *RSPH9*. Genetic results were confirmed by segregation analysis. The in-frame deletion of GAA in *RSPH9* has previously been published as pathogenic in both annotated *RSPH9* transcript variants (1 and 2). In contrast, the previously unpublished *RSPH9* frameshift deletion identified in KU-15.IV2 impacts only *RSPH9* transcript variant two. Regarding all 11 PCD individuals analyzed, IF results demonstrated absence of RSPH9 protein and TEM analysis showed the typical findings in *RSPH9* mutant individuals. Conclusions: We present the largest cohort of PCD individuals affected by the founder in-frame deletion GAA in *RSPH9*. This founder variant is the most common PCD-causing variant in Bedouin Arabs in Kuwait.

## 1. Introduction

### Genetics of Primary Ciliary Dyskinesia

Primary ciliary dyskinesia (PCD) (ORPHA:244) is a heterogeneous, autosomal recessive genetic disorder that is characterized by dysfunction of motile cilia and abnormal mucociliary clearance. PCD is characterized by respiratory distress syndrome, chronic bronchitis, bronchiectasis, chronic sinusitis, chronic otitis media, and male infertility. Chest imaging studies in PCD individuals reveal mainly atelectasis in the morphologic right middle lobe or lingua [1]. Approximately 50% of all PCD individuals have situs inversus or heterotaxy as a consequence of dysfunction of the motile node monocilia of the embryonic left–right organizer [2].^.^

PCD is an inherited disease caused by pathogenic variants in different genes, with more than 50 PCD-associated genes to date. Usually, PCD is inherited in an autosomal recessive manner, with both sexes being equally affected. A recent study has shown that the minimum global prevalence of PCD, calculated to be at least 1 in 7554 individuals, is likely underestimated (Hannah et al., 2022, [3]) and that the expected PCD frequency is higher in individuals of African ancestry than in most other populations. Moreover, certain groups that are isolated by geographical location show an increased occurrence of PCD, possibly due to endogamy, which is typical in the Kuwaiti population [3,4].

Despite progress in diagnosing PCD, only a limited number of patients with PCD receive a definitive diagnosis, which shows the restricted capacity for diagnosis of this pathology, with the first case being reported in the first decade of the 20th century. It is currently estimated that, even now, in 20–30% of individuals with phenotypic well-characterized PCD, no disease-causing genetic variants in any of the associated genes could yet be detected [5,6,7]. Overall, the diagnosis of PCD is still challenging, missed, or delayed since there is no “gold standard” test and some PCD variants might escape diagnostic criteria. For example, PCD variants caused by defects in radial spoke (RS) genes and/or genes associated with the central pair (CP) are frequently overlooked because they might escape diagnostic inclusion criteria for extended PCD diagnostics.

Radial spokes are T-shaped structures composed of head, neck, and stalk compartments and extend from the peripheral A-microtubule of the nine microtubule doublets toward the CP. RSs are involved in supporting the structure of the CP and in transmitting regulatory signals between the dynein arms and the CP [8]. Mutations in *RSPH1* [9], *RSPH4A* [10], *RSPH9* [10], *RSPH3* [11], *DNAJB13* [12], and *RSPH23/NME5* [13,14] cause radial spoke and/or ciliary central pair (CP) defects, which together with situs solitus account for a large proportion of PCD cases presenting with radial spoke (RS) and CP-associated defects [11]. However, these PCD variants are likely underdiagnosed.

Here, we present the largest previously undiagnosed cohort of PCD individuals affected by the founder inframe deletion GAA in the radial spoke gene *RSPH9.* This founder variant is the most common hereditary variant causing PCD in Kuwaiti patients belonging to different Arab tribes. We also provide evidence that a 2 bp frameshift deletion in *RSPH9* transcript variant 2 detected in trans with the founder inframe deletion, results in loss of function, shifting current classification from VUS to pathogenic. Our study extends previously published data from Castleman et al. on the prevalence of the founder homozygous 3 bp deletion in *RSPH9*, but also opens up the question: if *RSPH9* transcript variant 1 or *RSPH9* transcript variant 2 are relevant in respiratory cells, is the same genetic variant on genomic level predicted to result in either in-frame loss of the C-terminal Lys268 (p.Lys268del) or Glu286 (p.Glu286del) [10]?

## 2. Methods

### 2.1. Human Subjects

This investigation was undertaken with informed written consent from adult participants and the parents of children and under approval obtained from the Kuwaiti Ministry of Health Research Ethics Committee (Ethics ID: 62/2013). This consent covered taking blood samples from both patients and non-affected family members, as well as for the collection of nasal biopsies [15]. Family pedigrees were assembled using Cyrillic version 2.1 (http://www.cyrillicsoftware.com/, accessed on 11 September 2023 (Cyrillic 3)) according to the supplier’s guidelines. For most of the patients involved in the study, radiological data was collected with the aim of correlating genotype with clinical phenotype.

### 2.2. Genomic DNA and Exome Sequencing

Genomic DNA extraction from whole blood was achieved with the QIAamp mini-isolation kit (Qiagen, Hilden, Germany); concentrations were determined by UV spectrophotometry using a Nanodrop N1000 (Nanodrop Technologies Inc., Wilmington, DE, USA) [15]. Exome sequencing of genomic DNA was accomplished for all of the individuals marked with an asterisk in Figure S1. Target enrichment sequencing was performed, following manufacturer’s protocols, using SureSelect hybridization capture reagents with v6-capture (Agilent Technologies, Santa Clara, CA, USA). Enriched library preparations were sequenced on the HiSeq 2500 platform (Illumina, San Diego, CA, USA). Linkage analysis using exome data was performed using pipeline-produced variant call format (VCF) files.

### 2.3. Autozygosity Mapping and Variant Screening

**Figure 1:**
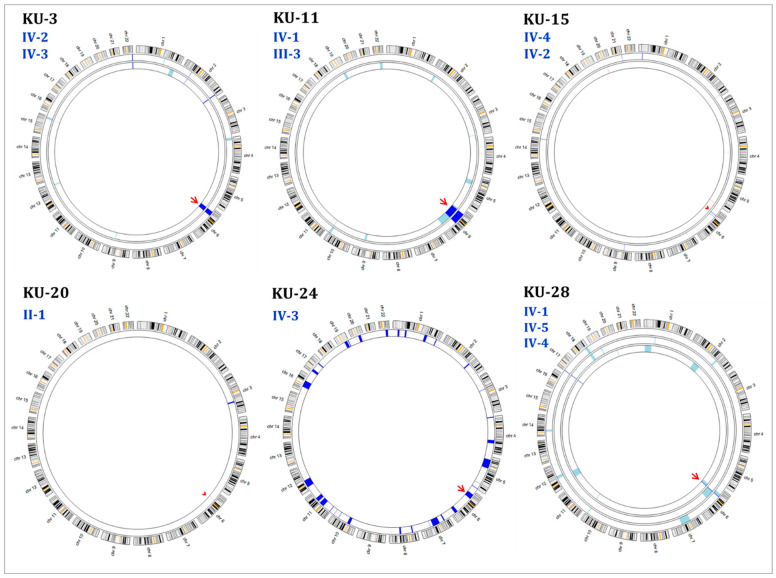
Summary of the Agile Multi Ideograms showing linkage scans for the 22-autosomal chromosomes for the affected individuals in each family with hereditary founder variant in *RSPH9*. Each ideogram represents the linkage scan as a circular axis for the PCD individuals belonging to the same family in one group. In multiplex families: the exclusive regions of homozygosity (ROH) shared between the affected relatives are displayed as navy blue bars. The homozygous regions that are not shared among affected relatives are displayed as light blue bars. In singleton families, all the detected ROH are displayed as navy blue bars since there is no shared ROH to be prioritized by the software, as seen for families KU-20 and KU-24. The shared ROH across the *RSPH9* locus at chr: 6 is indicated by a red arrow in each ideogram for families KU-3, KU-11, KU-24, and KU-28. Family KU-15 indicates that there are no detectable ROH regions for the IV-2 PCD individual, while the IV-4 patient has a minor shade at the gene locus (arrowhead) consistent with the genotype of the two PCD individuals. The linkage scan for KU-24, IV-3 shows a tremendous ROH for the PCD individual at several chromosomal segments and across *RSPH9* locus. However, the linkage for KU-20, II-1, PCD individual shows only one exclusive ROH at chr: 3 and a minor ROH interval (thin navy blue line) at the *RSPH9* locus at chr: 6 (arrowhead), which indicates that this founder variant has very high penetrance. Interestingly, the KU-28 family shows two small-scale ROH intervals shared among the three affected individuals IV-1, IV-4, and IV-5 (navy blue line) at chr: 6, across *RSPH9* locus, and they also have a shared IBD at chr: 17. There are multiple ROH that are not shared among the three affected individuals (light blue). In each ideogram, the linkage scan for each PCD individual belonging to the same family is presented from the ideogram edge (first circle) towards the center (last circle). The alignment is solely controlled by the software.

Genetic screening using autozygosity mapping was undertaken as previously described [15]. Initially, AgileMultiIdeogram software (version 3), enabled visualization of the homozygous intervals using exome data for linkage, in which all of the homozygous intervals are displayed against a circular ideogram for the 22-autosomal chromosomes for the related patients, as seen in Figure 1 [16]. The reference for genome annotation used was Human Genome Build hg19 (UCSC genome browser).

After that, multiple linkage analysis was performed for the eleven PCD individuals using AgileVCFMapper software (version 3), which is an Autozygous Variant Viewer application designed for determining the IBD intervals in each chromosome using the genotypes of exome data [16]. This analysis enables mapping of the haplotype for shared regions of autozygosity. Consequently, it was used successfully to determine the autozygous intervals harboring founder variants shared across multiple families by estimating the overlapping homozygous and concordant variants shared among unrelated affected individuals, as seen in Figure S2 [15].

Primers for variant confirmation and segregation analyses were designed using Primer3 software (http://frodo.wi.mit.edu/primer3/ (accessed on 11 September 2023)). The exon sequences were obtained from the University of California, Santa Cruz Genome Browser (http://genome.ucsc.edu/ (accessed on 11 September 2023)) for exon 6 of *RSPH9* gene. For the founder variant rs397515340, RefSNP Report—dbSNP—NCBI (nih.gov (accessed on 11 September 2023)) was used; the sequence of the forward primer used was “5′-GATTTGAACCAAGCCCTGAT-3′” and the sequence of the reverse primer used “5′-GCCAACATAGTGAAGCACCA-3′” to sequence the founder variant inherited in homozygous fashion in PCD individuals belonging to families (KU-3, KU-11, KU-20, KU-24, KU-28) and the KU-15.IV4 individual. For the compound heterozygous variants rs397515340 and rs760441659, inherited in heterozygous fashion in KU-15.IV2, another two sets of primers were used; for the founder variant, the sequence of the forward primer used was “5′-CCAGTGGAACCATAGCACCT-3′” and the sequence of the reverse primer used was “5′-TGAAGCTTAAGAAAATCATGTTTAGA-3′”. For sequencing the second heterozygous variant rs760441659, the sequence of the forward primer used was “5′-CCAGTGGAACCATAGCACCT-3′” and the sequence of the reverse primer used was “5′-TAGCACATTCCAACCCCTCT-3′”. The sequencing reaction was performed as previously described [15]. The Sanger sequencing results were analyzed using GeneScreen software [17].

### 2.4. Immunofluorescent Analyses of Nasal Biopsies

Samples of respiratory epithelial cells, obtained through nasal brush biopsy, were suspended in cell culture medium and spread onto glass slides, air dried, and stored at -80 °C until use. Cell samples from healthy control subjects and eleven PCD-affected individuals, highlighted with asterisks (Figure S1), were subjected to dual IF analysis with antibodies raised against axonemal components (visualized with red fluorescence) and antibodies to acetylated anti-tubulin (visualized with green fluorescence) as a ciliary marker. Cell nuclei were stained with Hoechst 33342 (blue fluorescence) [18]. Immunofluorescence images were captured with a Zeiss LSM 800 confocal microscope and further processed with ZEN and ImageJ software.

The panel of primary antibodies used for this IF screening included mouse monoclonal anti-tubulin (acetylated) antibody [Sigma Aldrich, St. Louis, MO, USA, T6793] to visualize ciliary microtubules and rabbit polyclonal antibodies to RSPH9 (1:300) [HPA031703, Atlas Antibodies, Bromma, Sweden]. The secondary antibodies employed were highly cross absorbed secondary antibodies: Alexa Fluor 488-conjugated goat antibodies to mouse (1:1000) [A11029, Molecular Probes, Eugene, OR, USA, Invitrogen, Waltham, MA, USA] and Alexa Fluor 546-conjugated goat antibodies to rabbit (1:1000) [A11035, Molecular Probes, Invitrogen].

### 2.5. TEM Analysis of Nasal Biopsies

For ultrastructural analysis of ciliated cells by transmission electron microscopy (TEM), nasal biopsy samples taken from the middle turbinate were firstly fixed with glutaraldehyde (2.5%) in Sorensen’s phosphate buffered (pH 7.4). After post-fixation, the samples were treated with osmium tetroxide (1.3%), dehydrated through graded ethanols, and immersed in hexamethyldisilazane, a drying reagent, before being infiltrated in embedding medium (1,2-Epoxypropan-Epon-mixture, 1:1) at 4 °C overnight. After polymerization, ultrathin sections were cut and picked out onto copper grids. The sections were stained with Reynold’s lead citrate. TEM was performed with a Philips CM10 electron microscope [18].

### 2.6. Validating the RSPH9 Founder Variant in Arab Population

The allele frequency of the identified variant was validated using real-time PCR by performing the allelic discrimination test using the same primers designed for Sanger sequencing as described above (Applied Biosystems, Waltham, MA, USA) [15]. In each experiment, positive (cases for which genotype was confirmed by Sanger sequencing) and negative (water) controls were included. A batch of an ethnically matched Arab control DNA panel collected from 100 normal subjects that belong to different Arabian tribes was run in parallel. Genotyping steps were performed in a 7500 Fast Real-Time PCR System according to the manufacturer’s instruction (Applied Biosystems).

## 3. Results

### 3.1. Autozygosity Mapping Reveals a Founder Homozygous Inframe Deletion in RSPH9

**Figure 2:**
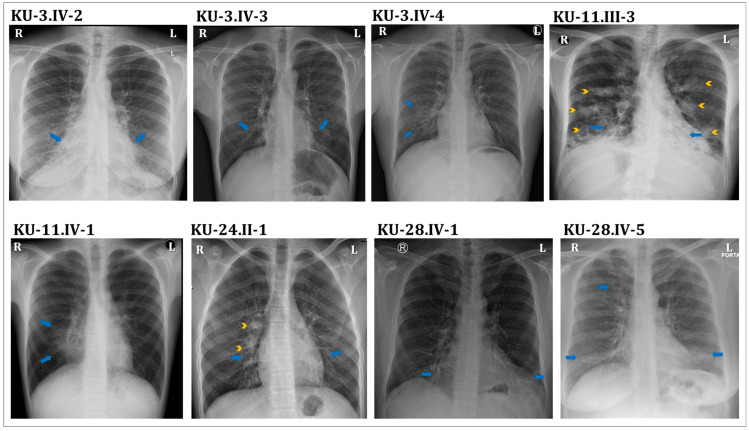
Radiological chest X-ray images for eight PCD individuals covered in this study. Chest X-ray of the KU-3.IV-2 patient showing mild diffuse bronchiectasis and patchy consolidations in bilateral lower zones, predominantly the retrocardiac regions (arrows), suggestive involvement of the right middle and the left lingular lobes. Chest X-ray of the KU-3.IV-3 patient showing dilated bronchi with “tram-track” appearance in the bilateral perihilar regions radiating into the lower zones (arrows). Chest X-ray of the KU-3.IV-4 patient showing subtle bronchiectatic changes with ill-defined hazy opacification in the medial right lower zone, suggestive of partial right middle lobe collapse (arrows). Chest X-ray of the KU-11.III-3 patient shows diffuse bilateral bronchiectasis with associated patchy opacities in all lung zones (arrowheads), representing secondary inflammatory changes (arrows). Chest X-ray of the KU-11.IV-1 patient showing central right perihilar bronchiectatic changes with an associated ill-defined opacity in the medial right lower zone causing obscuration of the right heart border, suggestive of complete right middle lobe collapse (arrows). Chest X-ray of the KU-24.II-1 patient showing dilated bronchi with “tram-track” appearance in the bilateral perihilar regions radiating into the lower zones (arrows). Small patches of consolidation are also seen in the right perihilar region (arrowheads), denoting infective changes. Chest X-ray of the KU-28.IV-1 patient showing mild diffuse bronchiectasis and patchy consolidations in bilateral lower zones, predominantly the retrocardiac regions (arrows), suggestive of involvement of the right middle and the left lingular lobes. Chest X-ray of the KU-28.IV-5 patient showing mild diffuse bronchiectasis and patchy consolidations in bilateral lower and the right upper zones (arrows). Situs solitus is seen in all patients.

PCD individuals were referred for genetic assessment to clinics run by the Ministry of Health in Kuwait. Most patients were suspected to carry hereditary variants, indicated by families having more than one affected individual with the same clinical phenotype. Individuals from six unrelated families were independently identified through PCD diagnosis in a cohort of fifty multiplex families recruited from different hospitals in Kuwait. These patients had hallmark clinical features characterizing the disease of PCD (OMIM:244400) that mainly included neonatal respiratory distress, chronic respiratory disease with symptoms of chronic airway infections, rhinosinusitis, otitis media, and bronchiectasis. All of the patients reported in this manuscript had normal positioning of the internal organs, i.e., “situs solitus”, which was assessed by chest X-rays (Figure 2). The ethnic background of all of the patients under this study is Asian Arab, belonging to different tribes from the Arabian Peninsula.

**Figure 3:**
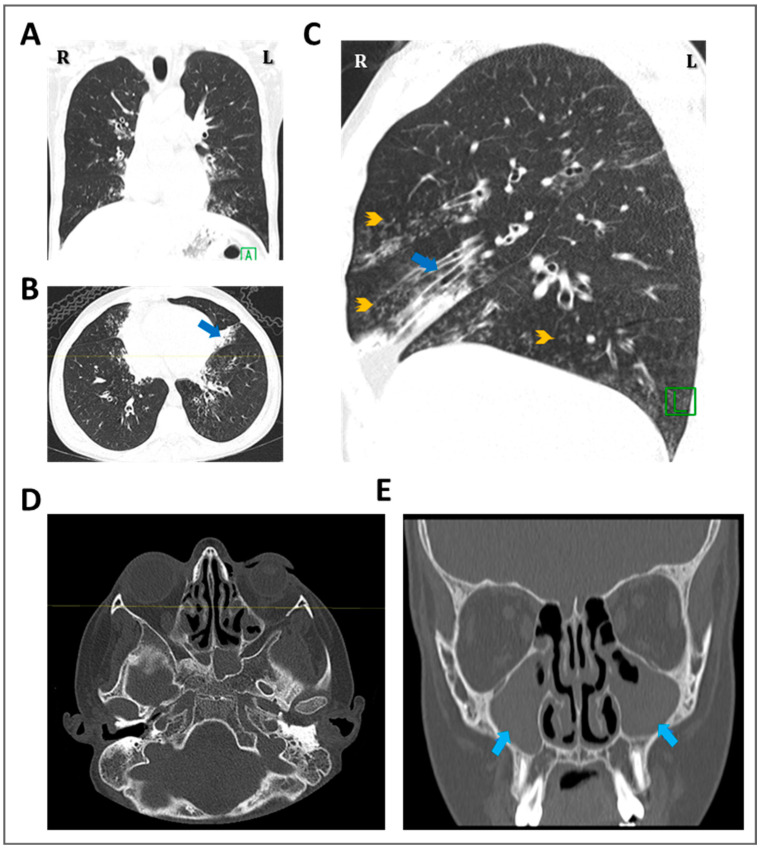
Radiological data for the KU-3.IV-3 patient shows the clinical morphology of the founder variant. Selected coronal (**A**), axial (**B**), and sagittal (**C**) recons of the high-resolution computed tomography (HRCT) chest study show mild diffuse bronchiectatic changes in both lungs, most notably in the lingular segments of the left upper lobe (arrows; blue), with extensive scattered nodular opacities showing “tree-in-bud” appearance (arrowheads; orange). These findings represent secondary infection in a patient with underlying chronic bronchiectatic changes. Selected axial (**D**) and coronal (**E**) images of a computerized tomography (CT) for paranasal sinus (PNS) study of the same patient show diffuse polypoid mucosal thickening opacifying both maxillary, ethmoid, and sphenoid sinuses, denoting chronic inflammatory PAN sinusitis changes.

This report summarizes the genetic analyses for these six unrelated families: KU-3, KU-11, KU-15, KU-20, KU-24, and KU-28, including a total of eleven PCD individuals. As seen in pedigrees (Figure S1), there are four multiplex families that have more than one affected individual. Radiological manifestations of PCD seen in our group of patients mainly included bronchiectasis and patchy consolidations or collapses, predominantly involving the middle and lower zone, as seen in Figure 2 and Figure 3. This is in contrast to defects in other radial spoke genes such as *RSPH1* that tend to associate with milder lung disease, situs solitus, and, occasionally, middle ear disease or hearing impairment [19].

According to the medical reports for the PCD individuals, the pediatric pulmonologists performed a differential diagnosis for almost all the patients and ruled out other conditions including asthma, cystic fibrosis, immunodeficiencies, and anatomic anomalies. Unfortunately, most of the signs and symptoms seen in these patients for upper and lower airway disease are also seen in healthy children. For this reason, the confirmed diagnosis of PCD is often made beyond childhood. The median age at which diagnoses in this study cohort were made was 5 years, with range of 4 to 14 years. The patients were hospitalized during infancy for recurrent respiratory distress and required oxygen administration. All of the patients reported the following symptoms: recurrent pneumonias, a chronic productive cough, wheeze, dyspnea, and constant nasal mucopurulent secretions. These symptoms gradually became more progressive with age. All individuals participating in our study have performed spirometry tests at different ages, mainly starting at ages 3–10. Nine individuals showed a decline in the pulmonary function with both obstructive and restrictive patterns, mainly seen at an older age (around 20 years of age). Two individuals (KU-15.IV-2, KU-15.IV-4) showed only an obstructive pattern and both are younger than 10 years of age.

None of the patients required surgical intervention for bronchiectasis. All of the patients underwent chest X-rays, seven cases underwent high-resolution computed tomography (HRCT) for chest, and in five cases computerized tomography (CT) for paranasal sinus (PNS) was performed. The representative chest X-ray images for eight PCD individuals support the clinical diagnosis of PCD, including consolidation and bronchiectasis with situs solitus (Figure 2). The HRCT studies showed clear evidence of bronchiectasis, including thickened bronchial wall in both lungs, most notably in the lingular segments of the left upper lobe (arrows), with extensive scattered nodular opacities as seen in the selected study for individual KU-3.IV-3 (Figure 3A–C). The CT-PNS studies showed chronic inflammatory pansinusitis in all patients, as seen in the selected study for individual KU-3.IV-3 (Figure 3D,E).

The initial linkage analysis was performed on all affected individuals belonging to the same family and these were analyzed together as one group, as shown in Figure 1. Mapping the shared identical by descent (IBD) intervals is the key for disease-gene identification in patients belonging to multiplex families. Consequently, IBD intervals were determined for each patient at the beginning of the study to estimate the shared IBD intervals, especially for closely affected relatives. The thickness of the IBD interval represents the extent of the autozygous regions [20,21]. For the multiplex families, the shared homozygous intervals, also referred to as autozygous intervals, were estimated. All of the families showed reliable linkage results and presented with a run of homozygous regions (ROH) across the *RSPH9* locus. Family KU-28 showed a minute shared IBD segment across the *RSPH9* locus in the three affected siblings (Figure 1).

Multiple linkage analysis showed a unique autozygous interval (blue bar) at chromosome 6 across the *RSPH9* locus that is shared among the ten PCD individuals (Figure S2). Interestingly, this unique autozygous interval can only be detected using a high resolution of linkage SNP-genotyping, as this shared IBD interval cannot be clearly visualized with AgileMultiIdeogram software in three patients (Figure 1, families KU-15 and KU-20), whereas it was detected using AgileVCFMapper software (Figure S2). Multiple linkage results suggested that a pathogenic variant in *RSPH9* is likely disease-causing in these families under study. All variants identified in exome data at the IBD segments that were found for the multiplex families in shared regions of homozygosity for the affected siblings were filtered. For singleton families, all IBD regions were considered for initial genetic screening. Genetic variants that were shared among affected relatives and located within IBD segments were precisely evaluated for pathogenicity for all the patients under study. In addition, screening for deleterious mutations in the currently more than 50 known PCD genes [22] was performed in parallel to rule out PCD-causing variants in additional previously published PCD genes.

Screening the variants of exome data at this unique IBD interval covering *RSPH9* revealed a homozygous three base pair deletion, which, using our bioinformatics pipeline, was annotated as [*RSPH9* (NM_001193341.2):c.856_858delGAA] in exon 6 of *RSPH9* transcript variant 2, predicting an in-frame deletion of the glutamic acid (p.Glu286del) in ten of these patients, consistent with previous linkage studies. On the genomic level, this variant is classified as NC_000006.11:g.43638659_43638661del (rs397515340).

**Figure 4:**
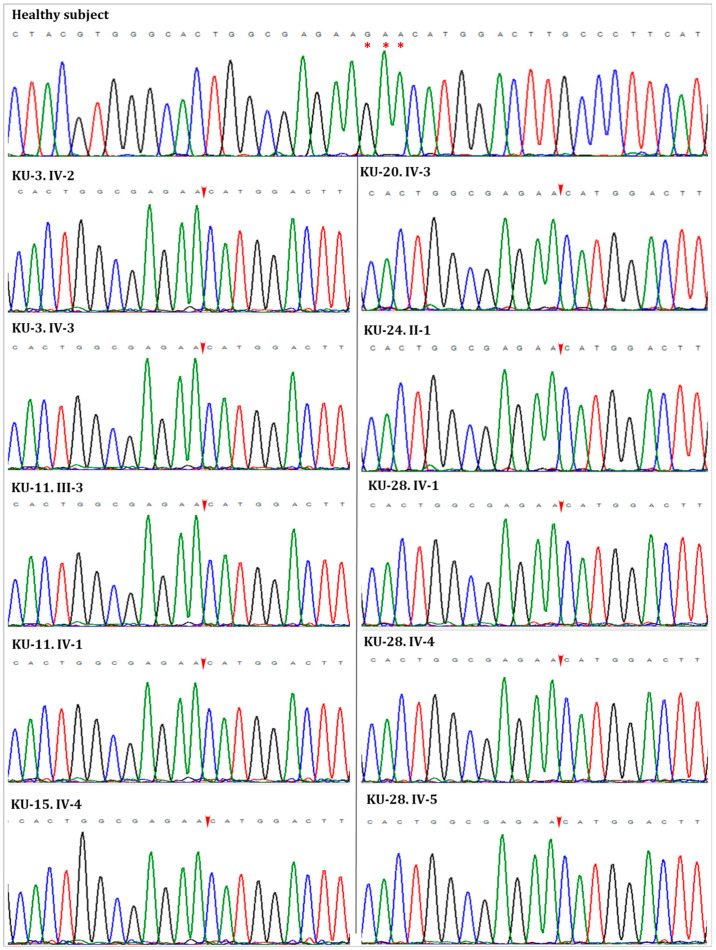
The sequencing chromatographs for the ten PCD patients versus control. All the patients with IBD across the gene locus have homozygous variant (c.856_858delGAA; p.Glu286del) in *RSPH9*. The position of the (GAA) deletion is indicated by three red asterisks in the healthy subject and by the red arrows in the chromatograms of PCD individuals.

Sequencing chromatograms for the ten PCD individuals confirms that all the patients are harboring the same homozygous in-frame deletion in *RSPH9* (Figure 4). Following the linkage and sequencing analyses, segregation analysis was performed to confirm that the identified variant in these ten PCD individuals segregates with the disease phenotype. Segregation analysis confirmed that this variant [*RSPH9* (NM_001193341.2):c.856_858delGAA] segregates with the disease phenotype and is the cause of PCD in the affected individuals (Supplementary Figures S3 and S4).

An allelic discrimination assay for this *RSPH9* in-frame deletion was performed using RT-PCR in order to estimate the carrier rate frequency of this variant [*RSPH9* (NM_001193341.2):c.856_858delGAA] using an Arab control panel composed of 100 DNA samples for normal individuals belonging to different Arabian tribes. As shown in Figure S6, the genotypes for the ten PCD individuals is homozygous for this in-frame deletion (GAA), and all their parents are heterozygous carriers for the wild type allele and the defective allele (*RSPH9*:c.856_858delGAA), which is consistent with Sanger sequencing data. Interestingly, the genotypes for the Arab control DNA panel show that all the healthy individuals have the two wild type alleles. This indicates that the variant is very rare, and the carriers are extremely scarce in Arab populations. This is consistent with our findings, since PCD is a rare genetic disease, and the carriers are rarely seen among healthy individuals (Figure S6).

### 3.2. Detection of Compound Heterozygous Variants in RSPH9 in KU-15.IV-2 Individual

In Family KU-15, the two individuals KU-15.IV-2 and KU-15.IV-4 present with classical PCD symptoms. As mentioned before, linkage analysis and exome sequencing have shown that individual KU-15.IV-4 carries the homozygous in-frame deletion [*RSPH9* (NM_001193341.2):c.856_858delGAA]. Remarkably, the linkage scan of individual KU-15.IV-2 shows a nonconcordant homozygous interval across the *RSPH9* locus (Figure S2, yellow lines). This type of linkage result is expected to be seen in patients having a homozygous non-founder variant or compound heterozygous variants, from which one allele presents a founder defective allele. WES analysis showed that KU-15.IV-2 carries two different heterozygous deletions in *RSPH9*. The first heterozygous variant is the three base pair in-frame deletion [*RSPH9* (NM_001193341.2):c.856_858delGAA] and has paternal inheritance. Notably, his affected cousin (KU-15.IV-4) is homozygous for this variant, and the fathers of these two PCD individuals are siblings (Figure S1). The second variant identified in KU-15.IV-2, a two base pair frameshift deletion [*RSPH9* (NM_001193341.2):c.915_916delAG; p.Arg305Serfs*42], has maternal inheritance and is only detectable in *RSPH9* transcript variant 2 (NM_001193341.2) because the position 915 exceeds the coding sequence lengths of 831 nucleotide acids of transcript variant 1 (NM_152732.5). Sanger sequencing confirmed that the two variants segregate with the disease phenotype (Figure S5) and are the cause of PCD in this individual.

### 3.3. Detection of Ultrastructural Defects of the Cilia

**Figure 5:**
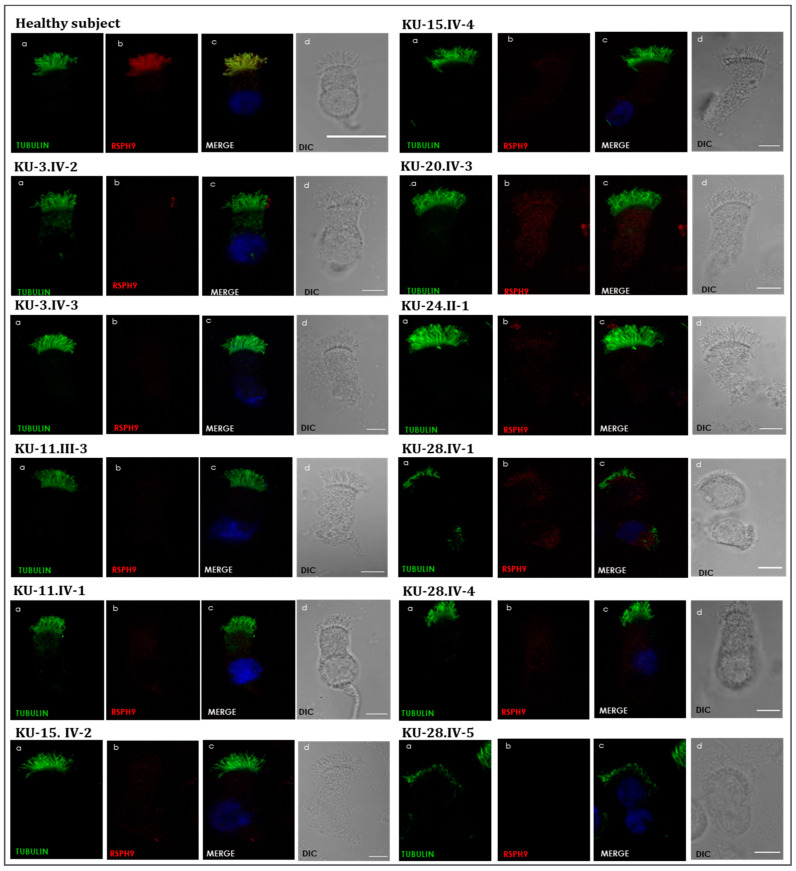
Immunofluorescence images of primary respiratory epithelial cells of PCD individuals versus healthy control sample. The IF was performed using monoclonal anti-acetylated α tubulin (panel a) and polyclonal anti-RSPH9 (panel b) antibodies. As seen in the healthy control sample, the merged images (panel c) show a yellow co-staining within the ciliary axoneme, which indicates that both proteins co-localized within respiratory cilia, compared with the images for PCD individuals that demonstrate an absence of anti-RSPH9 staining. (d) shows differential interference contrast figures (DIC) for analyzed cells, Scale bar is 10 μm.

**Figure 6:**
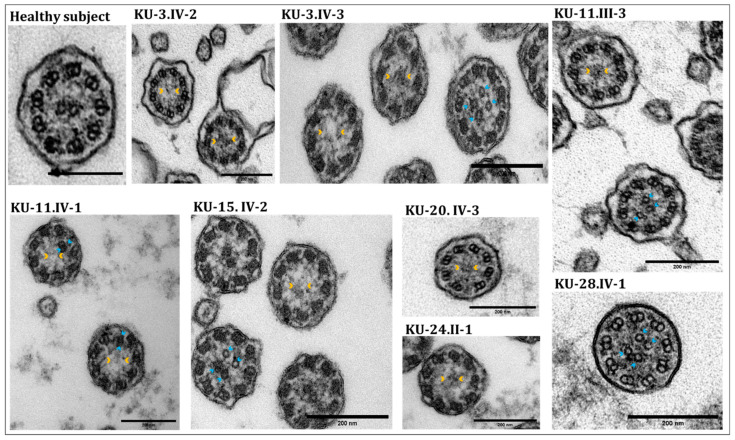
TEM images showing ultrastructural abnormalities of the ciliary axoneme for PCD patients. TEM results for ciliary axoneme of the epithelial cells from the respiratory system indicate that the PCD individuals mainly have an absence of central pairs (CP), as highlighted in arrowheads (orange), and mislocalization of the peripheral microtubule doublets (MDs) in some sections, as highlighted in arrowheads (blue). The ultrastructural ciliary defects indicate that the causative mutations in these patients affect the stabilization of the MDs and CP due to radial spoke defects consistent with their genotypes.

IF and TEM analyses were performed on nasal biopsies derived from all PCD individuals in this study to identify any structural abnormalities of the ciliary axoneme of respiratory epithelia. In the healthy subject sample, the yellow co-staining within the ciliary axoneme in merged images (Figure 5) indicates that both proteins co-localized within respiratory cilia [18]. In all patient samples, IF results showed negative staining for the corresponding RSPH9 protein in the cilia, indicating the absence of radial spoke components in all eleven PCD individuals, as seen in Figure 5. TEM analysis showed that the patients have either absence of central pairs (CP) or mislocalization of the peripheral microtubule doublets together with CP defects (Figure 6), consistent with previously published reports [10,23,24]. These IF and TEM analyses confirm that both the in-frame deletion [*RSPH9* (NM_001193341.2):c.856_858delGAA] and the frameshift deletion detected [*RSPH9* (NM_001193341.2):c.915_916delAG; p.Arg305Serfs*42] in KU-15.IV-2 are pathogenic and lead to loss of assembly of RSPH9 within the cilia (Figure 5 and Figure 6). These IF and TEM findings also provide evidence that, within respiratory cilia, RSPH9 transcript variant 2 is relevant.

## 4. Discussion

Autozygosity mapping is used for the discovery of autosomal recessive gene loci [25]. It is, in effect, a special form of homozygosity mapping that allows for mapping of the ancestral homozygous chromosomal segments that are identical by descent (IBD) and shared between affected relatives in consanguineous pedigrees. In addition, the same principle is applicable to map the deleterious founder variants from a cohort of unrelated patients with the same ethnic background. These types of patients are very common in Kuwait, as the structure of Kuwait society is tribe-based, rather than consisting of small unrelated families. Therefore, this mapping is efficiently used for multiplex families with more than one affected individual. Although the resolution of linkage, represented by the density of the “no-calls”, is not as powerful as SNP-genotyping linkage, autozygosity mapping has previously been used to precisely map two novel PCD-loci *LRRC6* and *CCDC103* genes in UK-Pakistani populations [26,27] and to identify a novel PCD gene, *CCNO*, that is associated with oligocilia and absence of situs abnormalities in a multiplex Kuwaiti family with four PCD-affected individuals [18]. It was also recently used to map a novel variant in *DNAI2* in seven PCD individuals belonging to a particular Arabian tribe in Kuwait [15]. Here, it was successfully used to detect an IBD interval across *RSPH9* locus at chromosome 6 and to identify the PCD-causing alleles in the affected individuals.

Using our approach, autozygosity mapping followed by WES analysis, we identified a homozygous in-frame deletion [*RSPH9* (NM_001193341.2):c.856_858delGAA] in the radial spoke head 9 homolog (*RSPH9*) gene in 10 out of 11 affected individuals from different tribes in Kuwait, originating from the Arabian Peninsula. This variant has previously been described as founder mutation in inbred Bedouin families and as geographically widespread in the Arabian Peninsula [10,23,24,28,29]. However, the original description refers to a different *RSPH9* transcript, transcript variant 1 (NM_152732.5), representing the shorter transcript encoding RSPH9 isoform 1 (NP_689945.2).

The officially annotated *RSPH9* transcript variant 2 (NM_001193341.2) uses an alternative in-frame splice site in the 5’ coding region of exon 3 and has an alternate exon in the 3’ coding region, which results in a frameshift compared to *RSPH9* variant 1 (NM_152732.5). The resulting protein RSPH9 isoform 2 (NP_001180270.1) is longer than the RSPH9 isoform 1 (NP_689945.2) and has a distinct C-terminus compared to RSPH9 isoform 1, but it lacks 15 amino acids encoded by exon 3 (Figure S7).

Taking these differences into account and according to currently annotated transcript reference sequences, the same genetic variant according to genomic localization (NC_000006.11:g.43638659_43638661del, rs397515340) has two different consequences on protein level, namely p.Lys268del if transcript variant 1 is used [*RSPH9* (NM_152732.5):c.804_806del] and p.Glu286del if transcript variant 2 is used [*RSPH9* (NM_001193341.2):c.856_858del]. However, in both cases, the variant is classified as pathogenic with very high penetrance and leads to loss of RSPH9 from the ciliary axoneme as shown by IF.

This variant is also the most common founder variant in the cohort of 100 PCD individuals in Kuwait, inherited in homozygous form in a total of 10 patients in our study cohort. This designates the rs397515340 variant as a hotspot variant in the Arab population in Kuwait, extending previously published studies [10,23,24,28,29] to the Kuwaiti population.

The eleventh patient inherited one founder variant allele and a second frameshift deletion [RSPH9 (NM_001193341.2):c.915_916delAG; p.Arg305Serfs*42], predicted to lead to premature termination of translation. However, this variant, according to Varsome [30], is classified as a variant of unknown significance (VUS) according to the criteria PVS1, defined as predicted loss of function variant, and PM2, defined as “absent from controls, or at extremely low frequency if recessive, in Exome Sequencing Project, 1000Genomes Project, or Exome Aggregation Consortium”. Here, we add two criteria to this variant interpretation: PM3, defined as “For recessive disorders, detected in trans with a pathogenic or likely pathogenic variant in an affected patient” and PS3, as two well-established functional tests, IF and TEM, confirmed loss of RSPH9 from ciliary axonemes in this patient. Consequently, (I) this frameshift variant has to be re-classified as pathogenic and (II) the function of *RSPH9* transcript 1 and transcript 2 in respiratory epithelial cells has to be evaluated in future studies.

All previous studies that reported on *RSPH9* in PCD individuals had difficulty in diagnosing PCD by TEM due to intermittent ultrastructural defects [10,31], suggesting that the majority of PCD individuals with mutations in *RSPH9* are misdiagnosed if TEM is used [10,31]. Here, in our study cohort, ciliary axonemes of all patient samples present with either absence of central pairs (CP) or mislocalization of the peripheral microtubule doublets together with CP defects, consistent with previously published reports. Loss of function of both identified genetic variants in *RSPH9* was also confirmed by IF, as all patient samples present with absence of RSPH9 from the ciliary axoneme. Thus, we provide independent functional evidence that both the founder in-frame deletion of GAA and the frameshift deletion unique for *RSPH9* transcript variant 2 are pathogenic, lead to loss of RSPH9 from the ciliary axoneme, and are the cause of PCD in the affected family members.

In summary, we present the largest number of genetically characterized PCD individuals with RS defects caused by loss-of-function variants in RSPH9 in consanguineous families from different tribes that originate from the Arabian Peninsula. Genetic testing is vital for PCD diagnostics of RS defects, as shown here for the founder in-frame GAA deletion in RSPH9, as this founder variant presents with high prevalence and high penetrance in our population. Thus, our findings will be beneficial for future screening of PCD individuals with situs solitus, especially those originating from the Arabian Peninsula. In addition, our data indicate that subsequent studies on alternative transcripts are needed to improve both genetic diagnostics and biological understanding.

## Data Availability

The data that support the findings of this study are available on request from the corresponding author, [D.A-M]. The data are not publicly available due to restrictions of Ministry of Health in Kuwait.

## Supplementary Materials

The following supporting information can be downloaded at: https://www.mdpi.com/article/10.3390/jcm12206505/s1, Figure S1: The pedigrees for the patients in this study. Figure S2: The Autozygosity mapping at chromosome 6 for the eleven patients with mutations in RSPH9. Figure S3: The chromatographs for the forward sequence traces for the seven parents under study. Figure S4: The chromatographs for the reverse sequence traces for the seven parents under study. Figure S5: The chromatographs for the members of Family KU-15 (KU-15.III-1, KU-15.III-2 and KU-15.IV-2. Figure S6: Allelic discrimination Plot for RSPH9 Gene Deletion. Figure S7: Schematic representation and sequences of RSPH9 transcript variant 1 (NM_152732.5; RSPH9-201) and 2 (NM_001193341.2; RSPH-202).
