# Supplementary material for: Mapping the Most Common Founder Variant in RSPH9 That Causes Primary Ciliary Dyskinesia in Multiple Consanguineous Families of Bedouin Arabs
Source: J Clin Med. 2023 Oct 13;12(20):6505. doi: 10.3390/jcm12206505 (PMC10607267; doi:10.3390/jcm12206505)
Supplement: Supplementary file 1 [file jcm-12-06505-s001.zip › jcm-2629742-supplementary.pdf]

Supplementary Figures

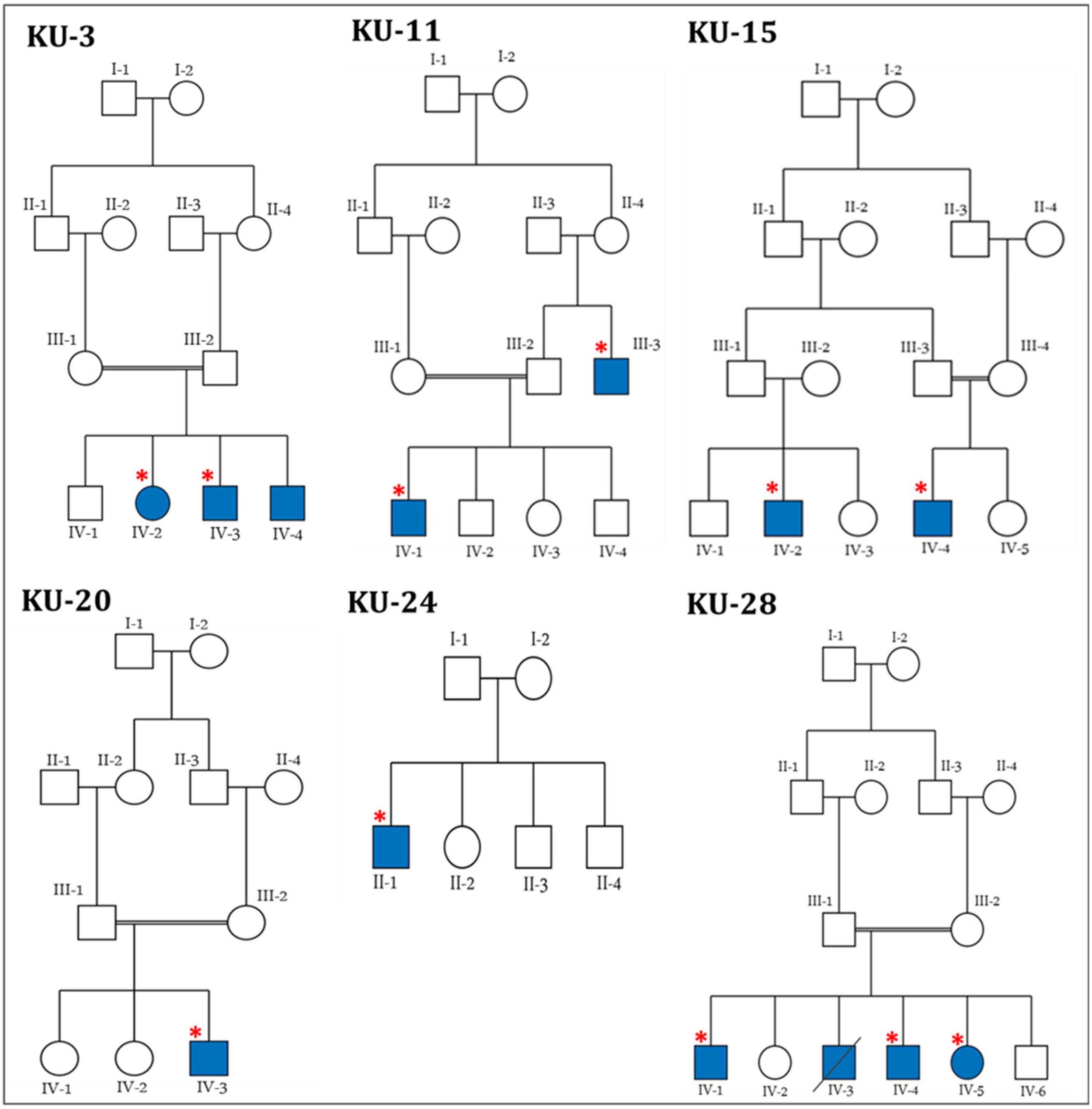

Figure S1: **The pedigrees for the patients in this study.** All the patients represented in the pedigrees have PCD symptoms with *situs solitus*. Family KU-3 is a multiplex family with three affected individuals. Family KU-11 is a multiplex family with two affected individuals (III-3 and IV-1), patient III-3 also has sterility with a history of multi-failure IVF. Family KU-15 is a multiplex family with two affected cousins IV2 and IV-4. Family KU-20 and KU-24 are singleton families that each has one affected male. Family KU-28 is a multiplex family with four affected siblings, all suffering from PCD, sterility for males and sub-sterility for the female. The DNA was taken from the patients highlighted with asterisks and their parents.

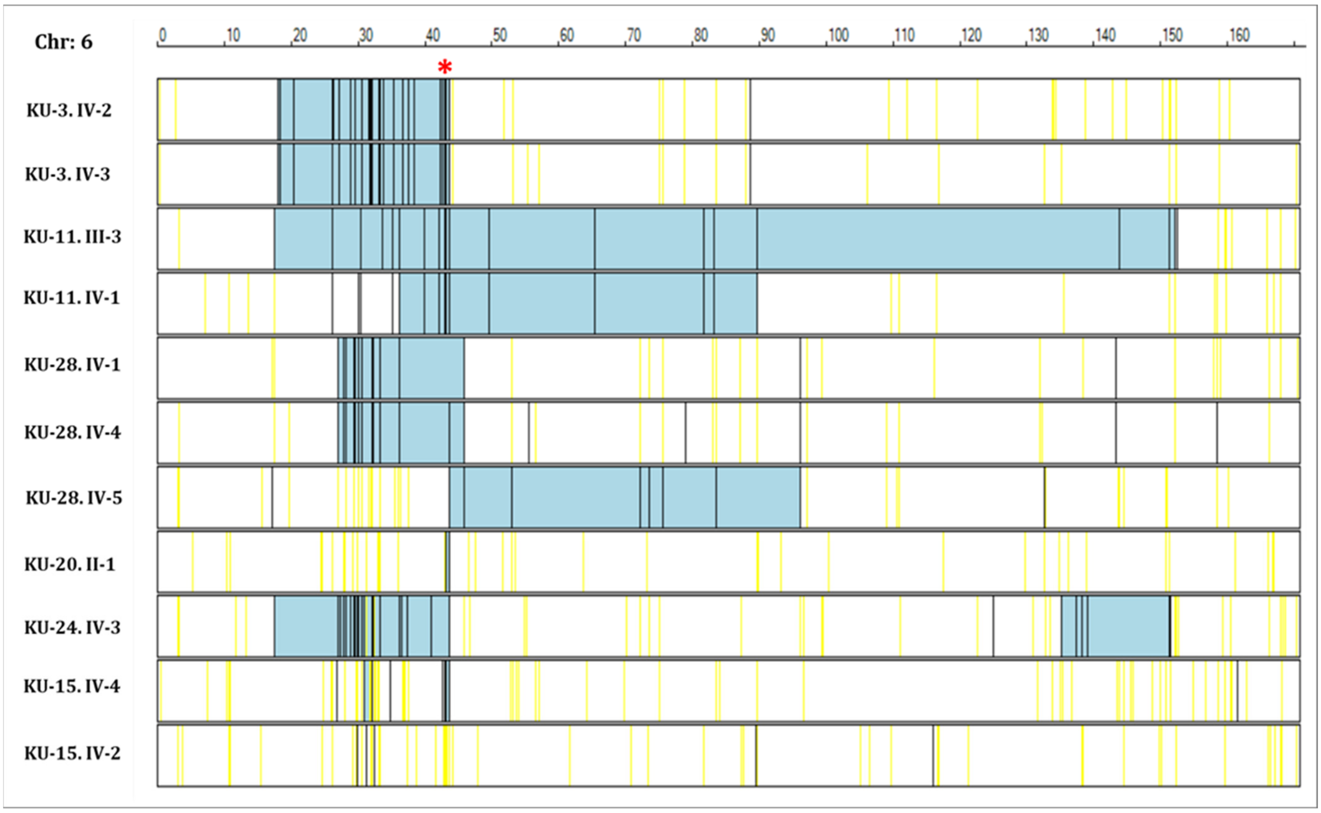

Figure S2: The Autozygosity mapping at chromosome 6 for the eleven patients with mutations in *RSPH9*. The autozygosity interval was determined using AgileVCFMapper software which is an Autozgyous Variant Viewer software. All the patients have a shared IBD region that highlighted by the asterisk and estimated to be (42,098,751-43, 926,528). The physical location of *RSPH9* is highlighted by the red asterisk. As seen, each row represents the genotypes for chromosome 6 for one PCD individual. The alignment of the chromosomal linkage data for each patient that is displayed in a separate row is totally controlled by the software which depends on the robustness of shared autozygosity interval that is detected in the 22 chromosomes. The linkage data is consistent with the sequencing results, since the ten patients with homozygous variant having IBD segment across the *RSPH9* locus (blue bar) and the eleventh patient with compound heterozygous variant KU-15. IV-2 has homozygous but not autozygous variants across the gene locus that represented as (yellow lines) and this is reasonable as

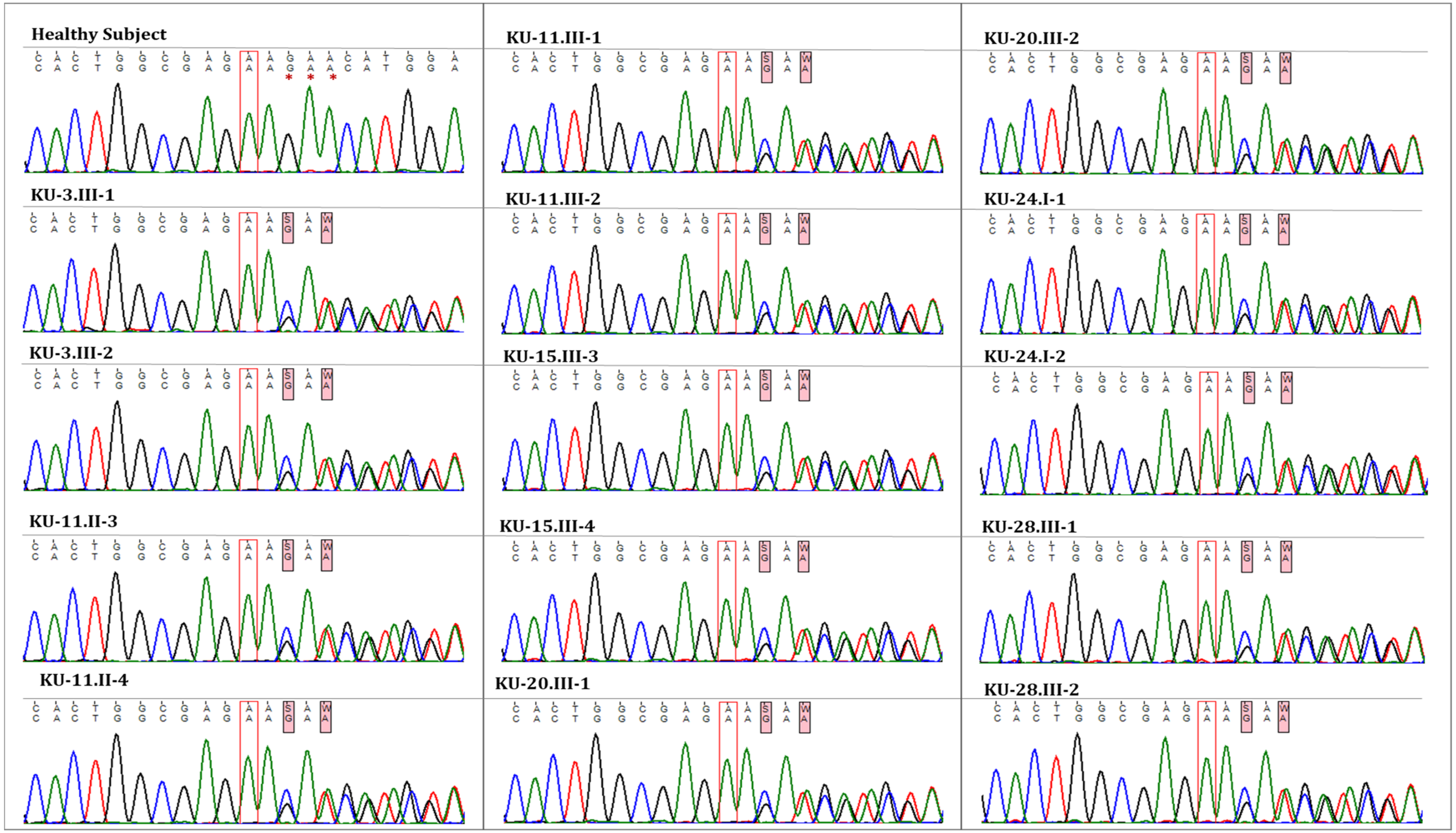

Figure S3: The chromatographs for the forward sequence traces for the seven parents under study. The position of the (GAA) deletion is indicated by three asterisks in the healthy subject. The forward sequencing results for the seven sets of parents demonstrate that all are carriers for the founder loss-of-function mutation (c.856_858delGAA) in exon 6 of *RSPH9*, which predicts a deletion of the Glutamic acid (p.Glu286del). This confirms that the mutation is segregated along with the disease phenotype in all the patients with homozygous deletion. The forward and reverse sequences were aligned at the same position by GeneScreen software that marked the adenine nucleotide which is the second nucleotide upstream of the deletion point as illustrated in sequence of the healthy subject.

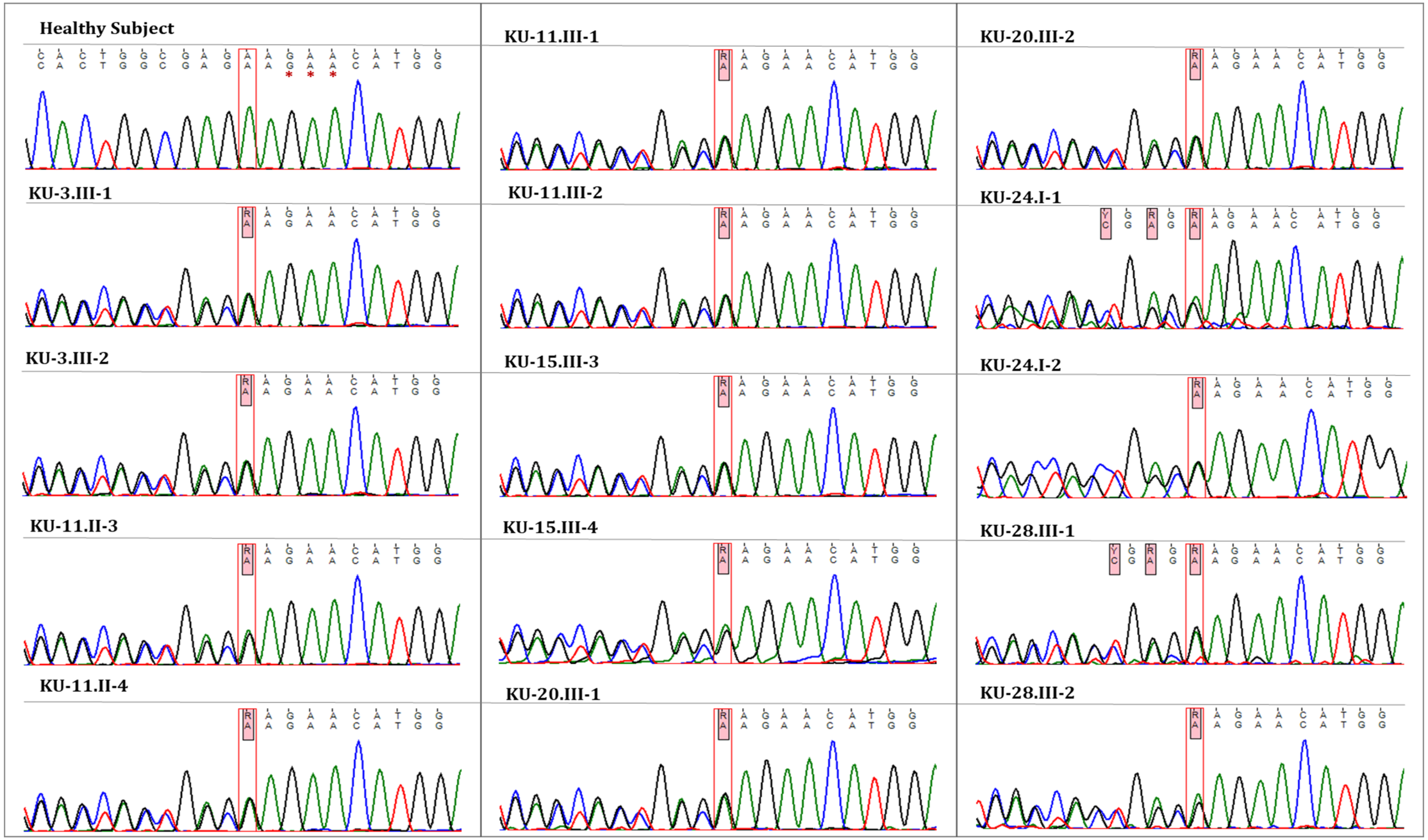

Figure S4: The chromatographs for the reverse sequence traces for the seven parents under study. The position of the (GAA) deletion is indicated by three asterisks in the healthy subject. The reverse sequencing results for the seven sets of parents also demonstrate that all are carriers for the founder loss-of-function mutation (c.856_858delGAA) in exon 6 of *RSPH9* which predicts a deletion of the Glutamic acid (p.Glu286del). This also confirms that the mutation is segregated along with the disease phenotype in all the patients with homozygous deletion. The forward and reverse sequences were aligned at the same position by GeneScreen software that marked the adenine nucleotide (red box) which is the second nucleotide located upstream the deletion point as illustrated in the sequence of the healthy subject. At this position, the heterozygous deletion for the reverse sequence is detected.

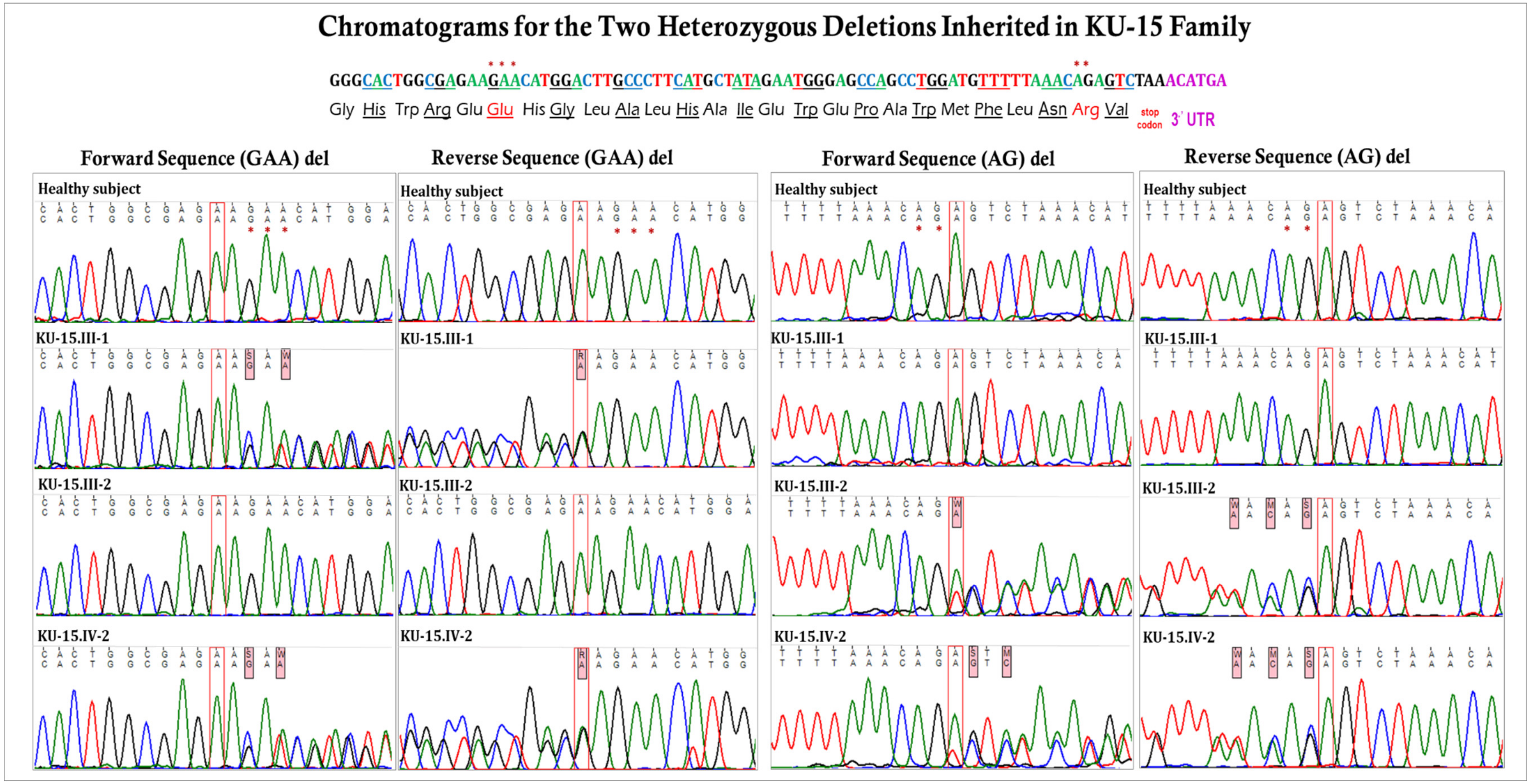

Figure S5: The chromatographs for the members of Family KU-15 (KU-15.III-1, KU-15.III-2 and KU-15.IV-2). Segregation analyses for the two heterozygous deletions (c.856_858delGAA; p.Glu286del) and (c.915_916delAG; p.Arg305Serfs*42) are presented. The position of the (GAA) and (AG) deletions are indicated by three asterisks and two asterisks, respectively in healthy subjects. A segment of *RSPH9* exon 6 sequence shows the positions of the two heterozygous deletions for illustrations. Segregation analyses for the two heterozygous deletions in the KU-15.IV-2 patient indicate that the compound heterozygous deletions are causing the disease phenotype. As seen in the figure, the forward and reverse sequencing chromatograms confirm that the first heterozygous deletion (c.856_858delGAA; p.Glu286del) has a paternal inheritance (KU-15.III.1), whereas the second heterozygous deletion (c.915_916delAG; p.Arg305Serfs*42) has a maternal inheritance (KU-15.III.2).

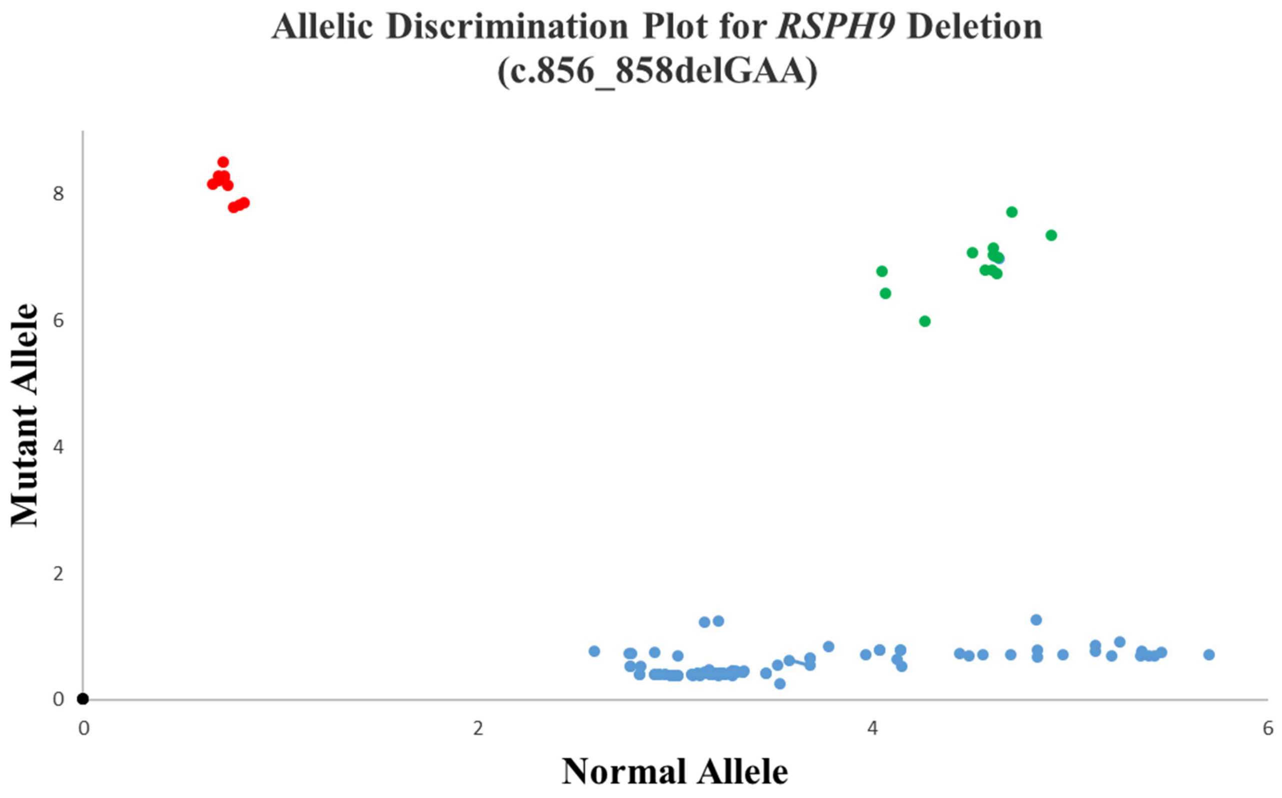

Figure S6: Allelic discrimination Plot for *RSPH9* Gene Deletion. All ten patients with the homozygous deletion showed homozygous mutant allele ($GAA^{-/-}$; red dots). The parents showed heterozygous mutant and normal allele patterns ($GAA^{-/+}$; green dots) consistent with Sanger sequencing data. A DNA panel composed of 100 DNA samples collected randomly from healthy Arab individuals from different areas in Kuwait were run as control panel in the assay. Results show that all of these controls carry the wild type allele in homozygous pattern ($GAA^{+/+}$; blue dots). This analysis indicates that this mutation is very rare and the carriers are rarely existing in the Arab population.

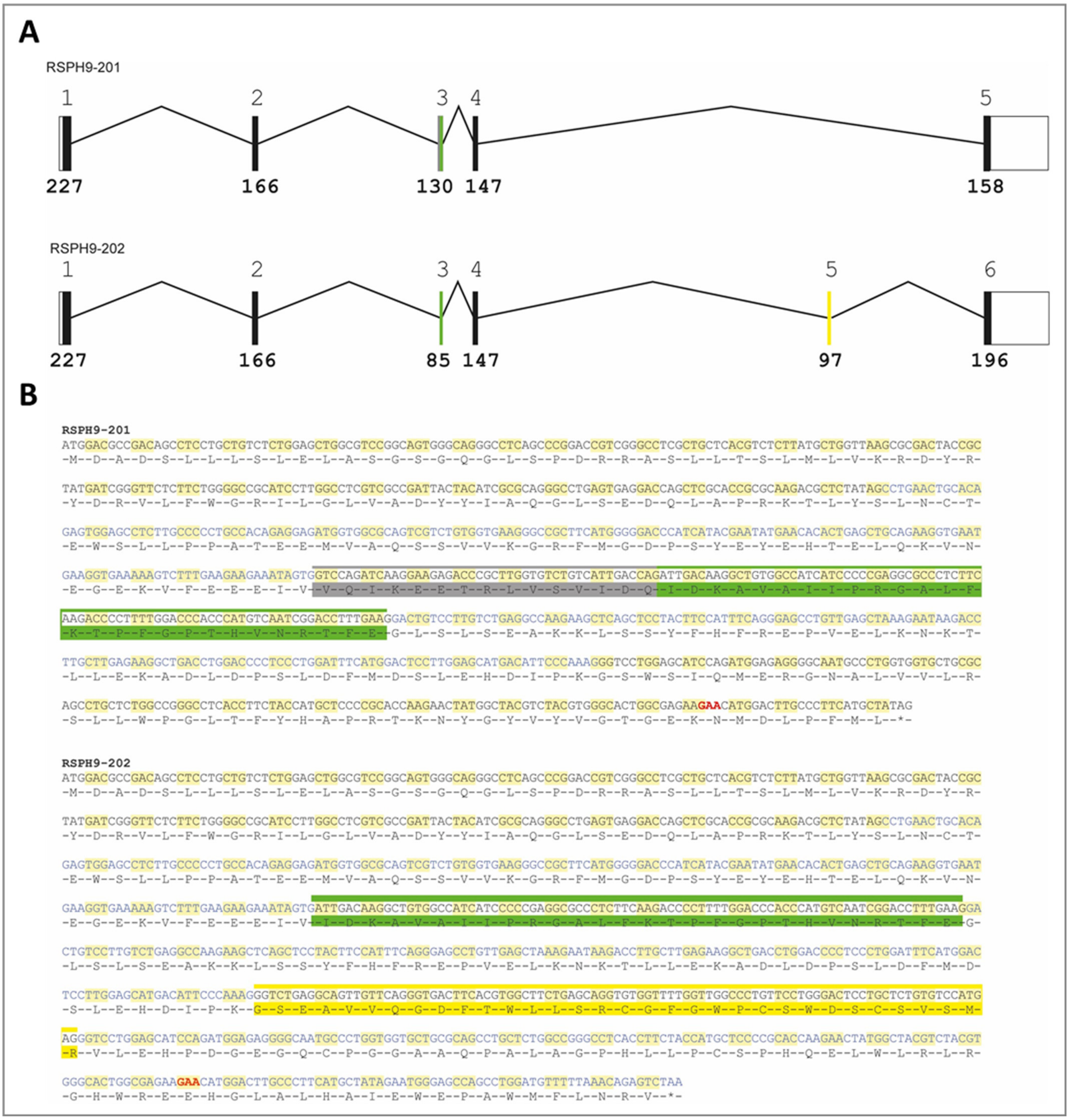

Figure S7: Schematic representation and sequences of *RSPH9* transcript variant 1 (NM_152732.5; RSPH9-201) and 2 (NM_001193341.2; RSPH-202). *RSPH9* transcript variant 2 (NM_001193341.2) uses an alternative in-frame splice site in the 5' coding region of exon 3 and has an additional exon in the 3' coding region. The resulting protein, RSPH9 isoform 2 (NP_001180270.1), is longer than RSPH9 isoform 1 (NP_689945.2). In addition, RSPH9 isoform 2 lacks 15 amino acids encoded by exon 3 and has a distinct C-terminus due to frameshift caused by the insertion of the additional exon. The 3 bp homozygous deletion of GAA in *RSPH9* is highlighted in both transcripts and will give p.Lys268del in transcript variant 1 and p.Glu286del in transcript variant 2.
